# The ReCoN intervention: a co-created comprehensive intervention for primary mental health care aiming to prevent involuntary admissions

**DOI:** 10.1186/s12913-022-08302-w

**Published:** 2022-07-19

**Authors:** Irene Wormdahl, Trond Hatling, Tonje Lossius Husum, Solveig Helene Høymork Kjus, Jorun Rugkåsa, Dorte Brodersen, Signe Dahl Christensen, Petter Sundt Nyborg, Torstein Borch Skolseng, Eva Irene Ødegård, Anna Margrethe Andersen, Espen Gundersen, Marit B. Rise

**Affiliations:** 1 Norwegian Resource Centre for Community Mental Health, NTNU Social Research, Trondheim, Norway; 2 Department of Mental Health, Faculty of Medicine and Health Sciences, Norwegian University of Science and Technology, Trondheim, Norway; 3 Centre for Medical Ethics, Institute for Health & Society, University of Oslo, Oslo, Norway; 4 Faculty of Health Sciences, Oslo Metropolitan University, Oslo, Norway; 5 Health Service Research Unit, Akershus University Hospital, Lørenskog, Norway; 6 Centre for Care Research, University of South-Eastern Norway, Porsgrunn, Norway; 7 Department of Health Care, Ullensaker Municipality, Ullensaker, Norway; 8 Department of Mental Health and Addiction, Nittedal Municipality, Nittedal, Norway; 9 Department of Mental Health and Addiction, Elverum Municipality, Elverum, Norway; 10 Department of Mental Health and Addiction, Porsgrunn Municipality, Porsgrunn, Norway; 11 Department of Mental Health and Addiction, Grimstad Municipality, Grimstad, Norway; 12 Mental Health Carers Norway Grimstad and environs, Grimstad, Norway; 13 Mental Health Norway Nittedal, Nittedal, Norway

**Keywords:** Involuntary admission, Severe mental illness, Mental health services, Primary care, Mental health recovery, Co-creation, Participatory research

## Abstract

**Background:**

Reducing involuntary psychiatric admissions is a global concern. In Norway, the rate of involuntary admissions was 199 per 100,000 people 16 years and older in 2020. Individuals’ paths towards involuntary psychiatric admissions usually unfold when they live in the community and referrals to such admissions are often initiated by primary health care professionals. Interventions at the primary health care level can therefore have the potential to prevent such admissions. Interventions developed specifically for this care level are, however, lacking. To enhance the quality and development of services in a way that meets stakeholders’ needs and facilitates implementation to practice, involving both persons with lived experience and service providers in developing such interventions is requested.

**Aim:**

To develop a comprehensive intervention for primary mental health care aiming to prevent involuntary admissions of adults.

**Methods:**

This study had an action research approach with a participatory research design. Dialogue conferences with multiple stakeholders in five Norwegian municipalities, inductive thematic analysis of data material from the conferences, and a series of feedback meetings were conducted.

**Results:**

The co-creation process resulted in the development of the ReCoN (Reducing Coercion in Norway) intervention. This is a comprehensive intervention that includes six strategy areas: [1] Management, [2] Involving Persons with Lived Experience and Family Carers, [3] Competence Development, [4] Collaboration across Primary and Specialist Care Levels, [5] Collaboration within the Primary Care Level, and [6] Tailoring Individual Services. Each strategy area has two to four action areas with specified measures that constitute the practical actions or tasks that are believed to collectively impact the need for involuntary admissions.

**Conclusions:**

The ReCoN intervention has the potential for application to both national and international mental health services. The co-creation process with the full range of stakeholders ensures face validity, acceptability, and relevance. The effectiveness of the ReCoN intervention is currently being tested in a cluster randomised controlled trial. Given positive effects, the ReCoN intervention may impact individuals with a severe mental illness at risk of involuntary admissions, as more people may experience empowerment and autonomy instead of coercion in their recovery process.

## Background

Reducing involuntary psychiatric admission is a global concern. The latest years increasing rates of involuntary psychiatric admissions in several countries give rise to growing concern [1]. For instance, from 2008 to 2017, countries like Australia, the United Kingdom, and the Netherlands reported an average annual percentage increase in rates of involuntary admissions by 3.44, 4.13, 4.17, and 5.18, respectively [1]. Geographical variations in rates of involuntary admissions are reported within and across countries [1, 2], indicating more use than necessary in some areas [2]. For instance, the rate per 100,000 people was 14.5 in Italy and 282 in Austria in 2015 [1]. Norway is among the countries that report relatively high numbers with a rate of 199 per 100,000 people 16 years and older in 2020 [3]. Comparison between countries can be challenging due to differences in legislation, health service organisation, and sociodemographic characteristics. However, these factors are found not to explain the substantial variations in the rates of involuntary admissions [1]. Involuntary admissions contradict the medical ethical principle of respect for individuals’ autonomy [4]. Service user organisations, as well as many national governments and international organisations such as the United Nations, have called for reductions in the use of involuntary admissions [5, 6]. Despite these efforts, rates in several countries have not decreased [1].

Aiming to prevent involuntary psychiatric admissions does not necessarily include an aim to prevent general psychiatric admissions. In this case, it is primarily the coercion phenomenon that aims to be reduced, not voluntary psychiatric admissions. Knowledge specifically on how to prevent involuntary admissions is thus needed. Studies of initiatives to prevent and reduce coercive practices in mental health settings worldwide show that some measures are effective [7]. For outpatient settings, shared decision-making interventions, like joint crisis plans and integrated care interventions, are among the measures that have shown effectiveness in reducing involuntary admissions [8]. However, in keeping rates of involuntary admissions low, experiences from the Trieste model indicate that a ‘whole system’ approach is more effective than individual measures [9], and comprehensive approaches have been found more effective than less comprehensive approaches in reducing seclusion and restraint in inpatient settings [10]. Six Core Strategies [11] and the High and Intensive Care model [10] are examples of such comprehensive interventions developed for inpatient settings that have been found effective [7, 10, 12–14]. Mental health care and treatment of people with severe mental illness (SMI), like schizophrenia or other psychotic disorders, are increasingly provided outside hospitals while people live in the community [15]. For many individuals with an SMI, primary health care provides the majority of services and might thus be in a key position to facilitate less restrictive services and prevent involuntary admissions. In addition, although involuntary psychiatric admissions in Norway are, as in most jurisdictions, effectuated at the secondary health care level, referrals to such admissions typically come from primary health care GPs and medical emergency services. Nonetheless, most research on preventing involuntary admissions has been aimed at secondary health care [7, 8] and comprehensive interventions developed for primary mental health care to reduce involuntary admissions are lacking.

In the latest years, the values of recovery orientation have increasingly been adopted as the framework for mental health service provision in many countries [16, 17]. Recovery-oriented services have a comprehensive approach, promoting citizenship, supporting individuals with SMI towards meaningful and productive lives, fostering hope that recovery is possible, and valuing individual autonomy [17, 18]. In this perspective, solutions to prevent involuntary admissions should include personal, relational, social, and contextual aspects relevant to the persons and services affected by them [19, 20]. Involving both persons with lived experience and service providers in the research process can ensure such aspects. Furthermore, it can enhance the quality and development of services in a way that meets different stakeholders’ needs [21, 22] and facilitate the adaption and translation of research into practice and enable implementation [21]. Thus, for this study, an action research approach with a participatory design [23, 24] was selected. Theoretically, the methodology relates to a systemic [21, 24] and social constructionist perspective [24], where knowledge generation is seen as context-sensitive and locally adaptive, and experiences and social relationships are central concerns [21, 24, 25]. In addition, dialogue, which acknowledges different forms of knowledge, is key to such action research [24].

Based on the lack of comprehensive interventions developed for primary mental health care, and in collaboration with multiple stakeholders, this study aimed to develop a comprehensive intervention for primary mental health care aiming to prevent involuntary admissions of adults.

## Method

Our co-creation process consisted of five dialogue conferences [26, 27] with multiple stakeholders, inductive thematic analyses of the data material created in the conferences, and feedback loops from stakeholders in a series of digital meetings. Dialogue conferencing is a method within action research that facilitates democratic dialogues and collaboration between stakeholders aiming towards future solutions and developments [26, 27].

A participatory research design with multiple stakeholders contributing during different phases requires a comprehensive description of how the study was performed to enable readers, reviewers and other researchers to assess the information and increase the study’s replicability [28]. Thus, to ensure the inclusion of all relevant information, our method description was guided by the “Template for Intervention Description and Replication” (TIDieR) checklist [28].

### Study setting

Norway is a high-income country with extensive publicly funded welfare services. Health care is provided at two levels. The primary care level is the responsibility of the 356 municipalities and includes general practitioners (GPs), medical emergency services, primary mental health and addiction services, rehabilitation, social care, (un) employment services and social housing services. Primary mental health care often provides long-term follow-up to persons with SMIs, commonly for years, and include services like sheltered housing, day-care facilities, therapeutic interventions, home nursing care, helping with practical tasks in the house, transport to doctor’s appointments, handling medications, and assisting with leisure activities. The secondary care level is the responsibility of the state. Here, four regional health trusts provide specialist health care through their regional psychiatric hospitals (inpatient treatment) and community mental health centres (community-based inpatient and outpatient treatment). There is a limited private sector with a few small institutions and some private practice psychiatrists/psychologists.

Regulated by the Norwegian Mental Health Act, the criteria for involuntary admissions are severe mental disorder, need for treatment, and/or risk to self or others [29]. Further, options for voluntary engagement should be exhausted or futile, and, unless there is a risk, a lack of the capacity to consent to treatment must be present [30]. Secondary mental health care holds the legal authority to decide and effectuate involuntary admissions.

This study forms part of the cluster randomised controlled trial (RCT) called Reducing Coercion in Norway (ReCoN), aimed at developing and testing a primary care level intervention to prevent involuntary admissions (ClinicalTrials.gov, NCT03989765). The present study was conducted in the five Norwegian municipalities constituting the intervention arm in the cluster RCT. To prepare for the co-creation of the intervention, qualitative interviews and focus groups were performed to explore individuals’ paths towards referral to involuntary admissions [31] and current practice in the municipalities [32]. The ReCoN trial did not provide finances to the participating municipalities. Thus, the measures to be included in the co-created intervention had to be feasible within current services’ existing resources.

### Design of the co-creation process

Five municipalities had volunteered to take part in the development of the intervention and the subsequent effectiveness testing. Stakeholders from primary and secondary mental health services, primary medical services, police, and users and carers’ advocacy organisations from these municipalities were invited to participate in the co-creation process, which consisted of 1) five one-day dialogue conferences [26, 27], one in each municipality, where multiple stakeholders worked together to suggest, discuss, and prioritise measures for the intervention; 2) inductive thematic analysis [33] of the suggested and prioritised measures presented on posters from the five dialogue conferences; 3) a series of dialogue meetings with feedback loops and discussions concerning intervention drafts based on steps 1 and 2.

### The one-day dialogue conferences

The one-day dialogue conferences were held in February and March 2020, and all followed the same structure with brief theoretical lectures and three group work sessions.

First, the overall research project and preliminary results from the mapping of practice were presented. The preliminary results comprised the main themes, 1) follow-up of individuals, including the use of plans/tools, 2) primary care service development, 3) housing/living conditions, 4) employment/activity, 5) social network/loneliness, 6) staff competence training, 7) collaboration between services at primary and secondary care level, 8) user and carer involvement and training.

Second, in the first group work session, as far as possible, stakeholders from the same service/organisation formed the groups to facilitate security and confidence for all participants to participate in the dialogue and share suggestions for measures. The groups were instructed to have a brainstorm, suggesting all potential measures and writing them on a piece of paper. They were given both blank Post-it notes, which they could fill in themselves and a set of pre-completed notes with suggestions from the preliminary results of the mapping of current practice. They were free to use or not to use the pre-completed notes. At the end of the session, the participants distributed all their suggested measures on posters representing the eight main themes from the preliminary results of the mapping of current practice.

Third, a brief theoretical lecture about Six Core Strategies [11] was given as an example of the implementation of complex interventions.

Fourth, for the second group work session, new groups were formed. Here, as far as possible, multiple stakeholder groups were represented in each group to ensure a broad perspective in the further dialogue and the prioritising of the measures from group work session one. Each group got posters of two main themes and collaborated on prioritising the suggested measures down to a maximum of ten. They were instructed to emphasise that the measures were feasible and realistic within current practice. In addition, they were asked to concretise measures that were not specific enough.

Fifth, in the third group work session, the group participants remained the same as in the second group work session while the main theme posters rotated between the groups. The groups were instructed to prioritise the ten remaining measures from one to ten, based on which measures they thought were most important to include in the intervention.

Sixth, all participants took part in what was called “the star round”. Here, the posters with the prioritised measures were hung on the wall for everyone to see. All participants got three stick-on stars that they were asked to place behind the measures they thought were the most important ones to include in an intervention for primary care level aimed to reduce the use of involuntary admissions. They could place each star at a different measure or use two or three stars for one measure they thought was particularly important.

### Analysis

Inductive thematic analysis [33] of the prioritised measures across all five municipalities was performed by the research team in March 2020.

First, each measure was written on a piece of paper that physically was used to sort measures back and forth into emerging categories during the analytical process. This allowed the researchers to stay close to the data and facilitated an inductive development of the intervention.

Second, the measures in each category were sorted based on how they had been prioritised. Measures with high priority in several municipalities were kept, while others, which held high priority in one municipality and low priority or were not included in others, were removed.

Third, the remaining measures in each category were sorted into sub-categories, constituting strategy areas (categories) with action areas (sub-categories) and measures for the intervention.

### Feedback from stakeholders

A total of eight two-hour digital video meetings with key stakeholders were held from May to September 2020. It was four meetings with managers from the municipalities and four meetings with persons with lived experience (two meetings) and family carers (two meetings). The feedback meetings had the following structure:

First, the research group prepared drafts of one or two strategy areas with their respective action areas and measures before each of the four feedback meetings with the managers. The drafts were e-mailed to the participants before the meetings. Relevant literature was incorporated into the description of the strategy areas.

Second, the participants gave oral feedback on the included measures. Some of the participants also gave written feedback after the meetings. In their feedback, the managers particularly emphasised whether the measures were specific and realistic to implement within the current practice during their first implementation year.

Third, in the feedback meetings with the representatives from the advocacy organisations, they gave feedback on drafts of all the strategy areas in the first meeting. In the second meeting, they gave feedback on the associated intervention tools, with particular emphasis on the measures being positive and not experienced as a violation, stigmatising, or having other potential adverse effects for individuals or their family carers, in the second meeting.

Finally, following the participants’ feedback, the research group revised the intervention and wrote a descriptive manual to inform implementation.

We originally planned for the feedback in the third phase of the process to occur in a sixth dialogue conference, in which participants from all five municipalities together determined the final intervention. Due to the Covid-19 pandemic and the lockdown in Norway in spring 2020, this had to be cancelled. Instead, in collaboration with stakeholders, the third phase was redesigned as four two-hour digital meetings with two or three key persons from each municipality. Also, two digital meetings each were held with representatives from the advocacy organisations of Mental Health Norway and Mental Health Carers Norway.

### Participants and recruitment

A total of 117 persons from multiple stakeholder groups participated in five dialogue conferences, one in each municipality, with stakeholder groups and sample distribution shown in Table 1. With a resource limitation of fifty participants at each dialogue conference, the participants were strategically recruited to include multiple stakeholder groups representing experiences from various services, lived experience, and family carers. The distribution among stakeholder groups aimed for more than half of the participants to come from primary mental health care since they were the services to which the intervention was aimed to be adapted. Further, it was desired that approximately one-fifth of the participants represented persons with lived experience and family carers to get multiple experiences and empower them as stakeholder groups in the co-creation process. Inclusion criteria were 1) working in relevant services in the actual municipalities or collaborating specialist mental health services, 2) individuals with lived experience of SMI and/or involuntary admission, or 3) family carers of individuals with lived experience of SMI and/or involuntary admission. The professional participants were recruited through service managers. Persons with lived experience and family carers were recruited through the local groups of the advocacy organisations Mental Health Norway and Mental Health Carers Norway. All participants registered for the dialogue conferences digitally. The register form included a check box section where participants consented to participate in the study. Four or five of the researchers, who have various clinical and research backgrounds, including a peer researcher, participated in each dialogue conference as facilitators and lecturers.

**Table 1:** Participants at dialogue conferences distributed by stakeholder groups and municipality

Municipality	A	B	C	D	E	Total
Stakeholder groups						
Managers primary mental health service	4	2	7	2	4	**19**
Staff primary mental health service	13	8	12	7	5	**45**
Secondary mental health service	4	1	4	4		**13**
Primary healthcare medical practitioners^a^	2	1	2	2		**7**
Police	2		2	1		**5**
Other primary level services^b^				2	5	**7**
Persons with lived experience	1		1	4	3	**9**
Family carers	3	1	1	1	1	**7**
Students in primary health services	3			1		**4**
Police student	1					**1**
**Total**	**33**	**13**^**c**^	**29**	**24**	**18**^**c**^	**117**

^a^Primary health care medical practitioners include chief municipal medical officers, general practitioners (GPs), and medical emergency services (doctors/nurses)

^b^Other primary level services include social welfare services, housing services and municipal purchaser offices

^c^Dialogue conferences in municipalities 2 and 5 were due just a few days before the Covid-19 pandemic lockdown in Norway in March 2020 and thus had some last-minute cancellations from health care staff redirected to crisis management and other clinical tasks

A total of 12 persons from the primary mental health services – two or three from each municipality – participated in the digital meetings and provided feedback to the research group after analysis. Nine were primary mental health services managers, one was a psychologist, and two were project managers/service development managers. Four of the researchers participated in each meeting. We held a total of four digital meetings with persons with lived experience (two meetings) and family carers (two meetings). Four persons from the advocacy organisation Mental Health Norway and three from Mental Health Carers Norway participated, along with two of the researchers.

## Results

**Fig. 1:**
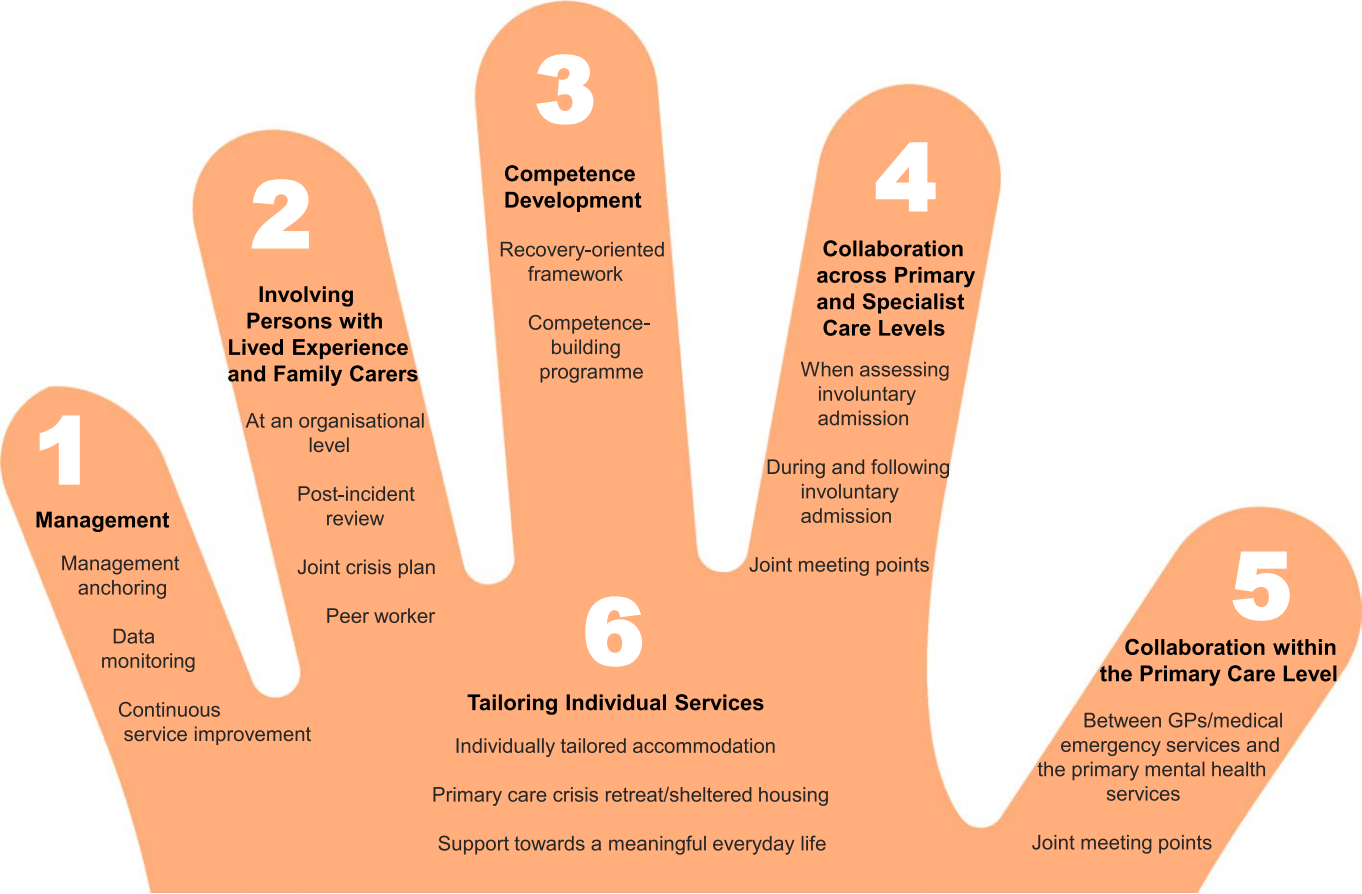
The ReCoN intervention: Strategy areas and associated action areas (Hatling T, Husum TL, Kjus SHH, Wormdahl I. [The ReCoN intervention. Strategies to reduce involuntary admissions]. Trondheim: Norwegian Resource Centre for Community Mental Health; 2020) (Reproduced with permission from the Norwegian Resource Centre for Community Mental Health, NTNU Social Research)

The co-creation process resulted in an intervention with six strategy areas: 1) Management, 2) Involving Persons with Lived Experience and Family Carers, 3) Competence Development, 4) Collaboration across Primary and Specialist Care Levels, 5) Collaboration within the Primary Care Level, and 6) Tailoring Individual Services. The work associated with the strategy areas was intended to be concurrent, not sequential. Each strategy area has two to four action areas (see Fig. 1), which in turn have a number of measures that constitute the intervention’s practical actions or tasks, as listed in Tables 2, 3, 4, 5, 6 and 7. These figures and tables are translated from the manual, developed in Norwegian, to guide implementation [34]. We also developed an implementation workbook to note specifications and plans for the implementation of the different measures.

**Table 2:**
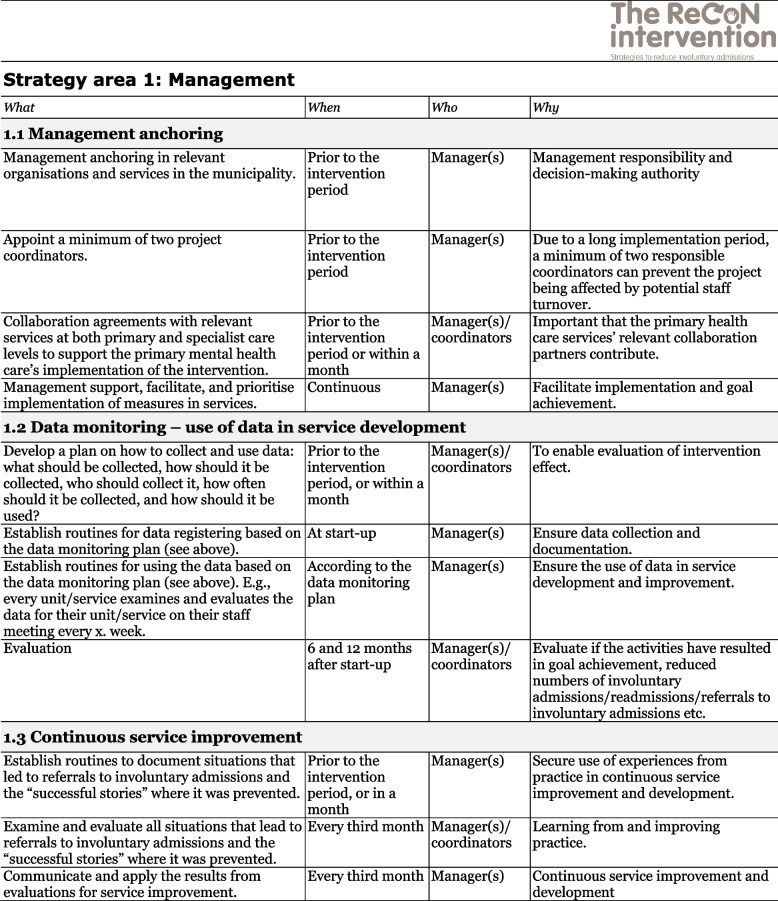
Strategy area 1: Management

This table is translated from the Norwegian manual (Hatling T, Husum TL, Kjus SHH, Wormdahl I. (2020). [The ReCoN intervention. Strategies to reduce involuntary admissions]. Trondheim: NAPHA - Norwegian Resource Centre for Community Mental Health.)

**Table 3:**
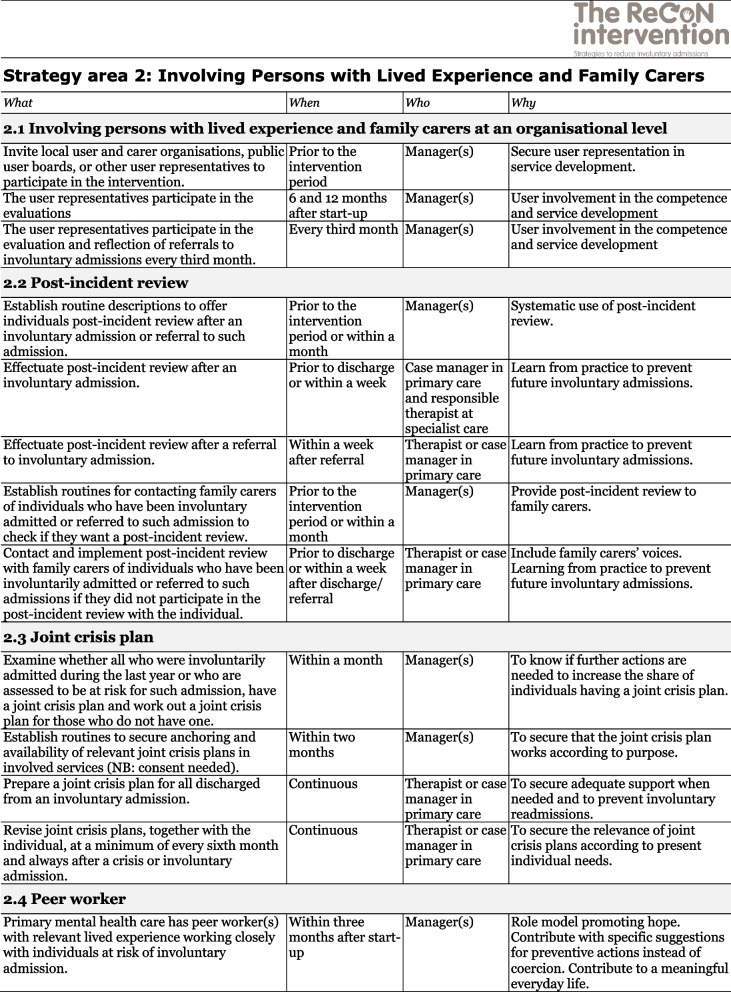
Strategy area 2: Involving Persons with Lived Experience and Family Carers

This table is translated from the Norwegian manual (Hatling T, Husum TL, Kjus SHH, Wormdahl I. (2020). [The ReCoN intervention. Strategies to reduce involuntary admissions]. Trondheim: NAPHA - Norwegian Resource Centre for Community Mental Health.)

**Table 4:**
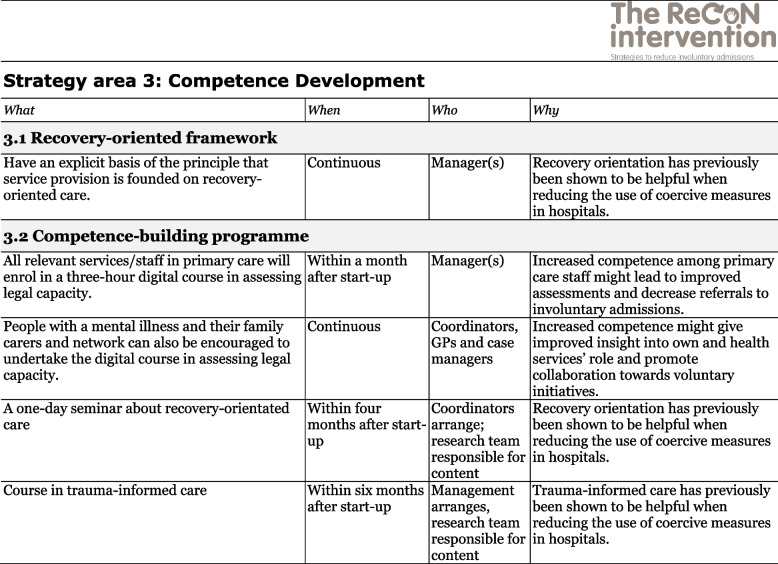
Strategy area 3: Competence Development

This table is translated from the Norwegian manual (Hatling T, Husum TL, Kjus SHH, Wormdahl I. (2020). [The ReCoN intervention. Strategies to reduce involuntary admissions]. Trondheim: NAPHA - Norwegian Resource Centre for Community Mental Health.)

**Table 5:**
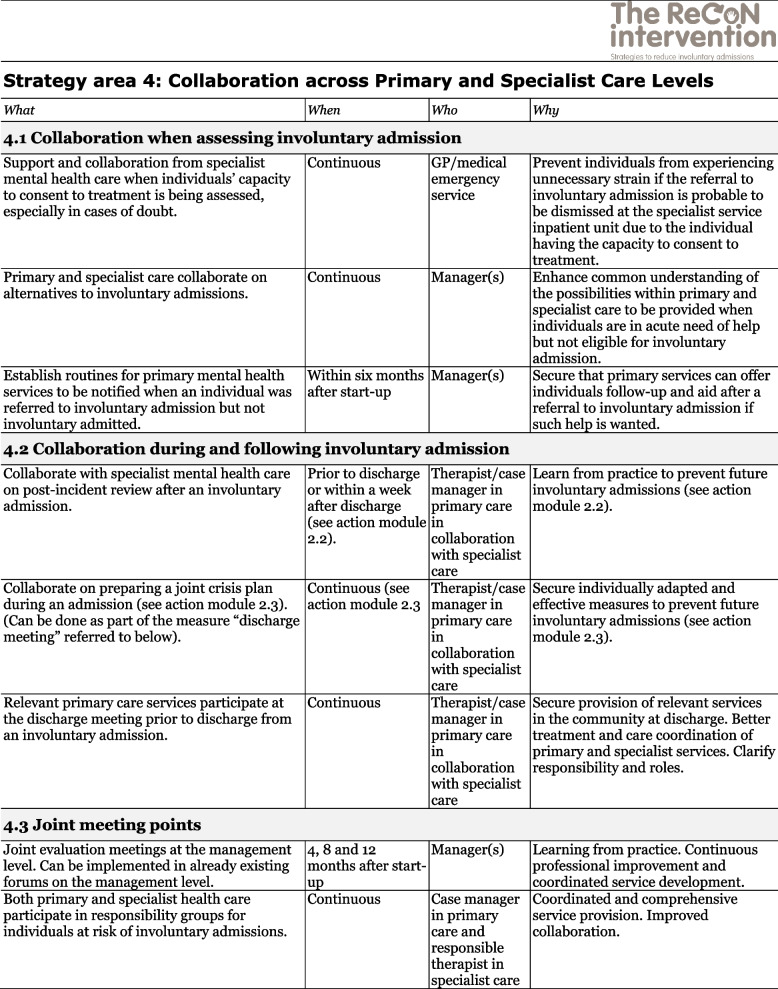
Strategy area 4: Collaboration across Primary and Specialist Care Levels

This table is translated from the Norwegian manual (Hatling T, Husum TL, Kjus SHH, Wormdahl I. (2020). [The ReCoN intervention. Strategies to reduce involuntary admissions]. Trondheim: NAPHA - Norwegian Resource Centre for Community Mental Health.)

**Table 6:**
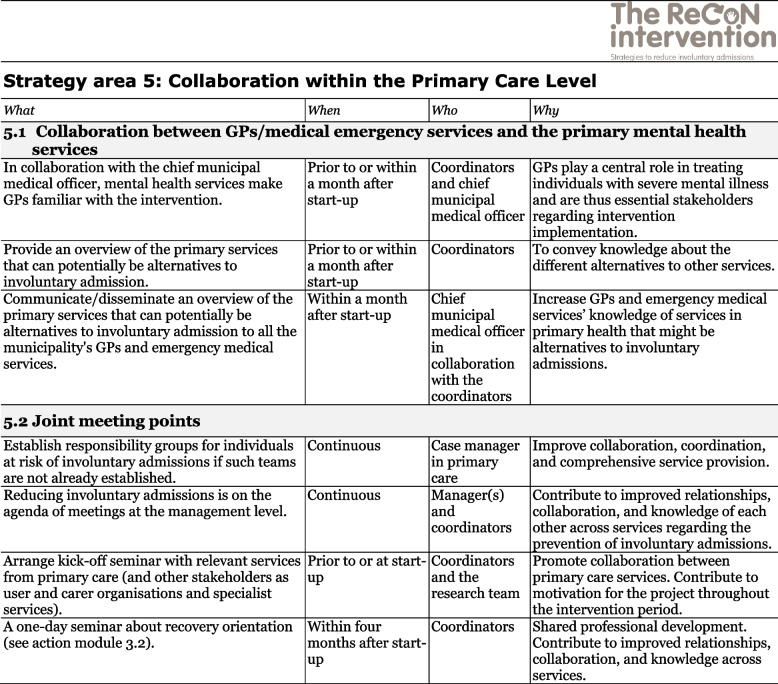
Strategy area 5: Collaboration within the Primary Care Level

This table is translated from the Norwegian manual (Hatling T, Husum TL, Kjus SHH, Wormdahl I. (2020). [The ReCoN intervention. Strategies to reduce involuntary admissions]. Trondheim: NAPHA - Norwegian Resource Centre for Community Mental Health.)

**Table 7:**
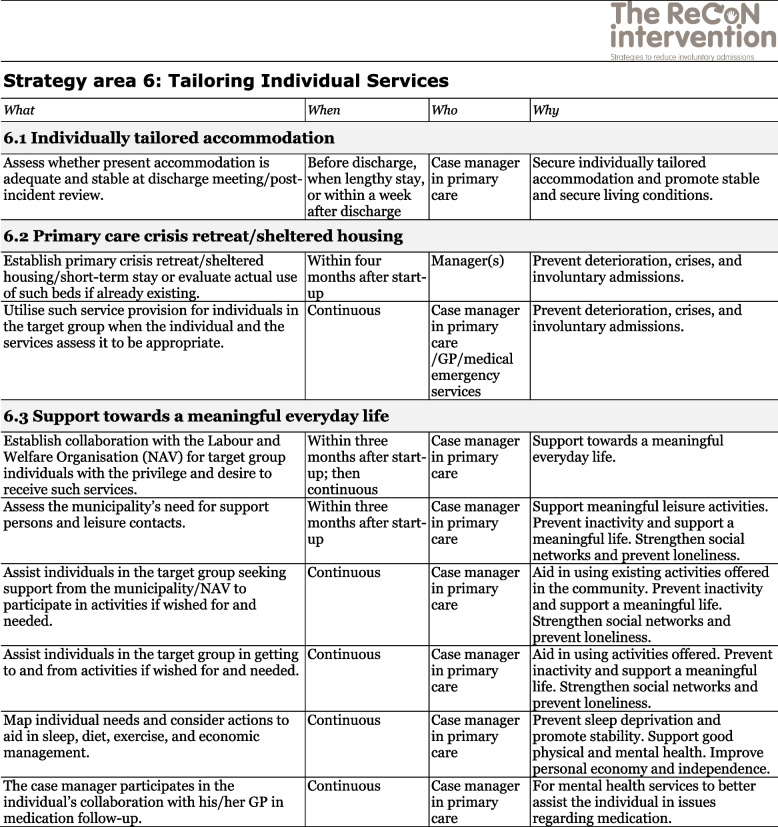
Strategy area 6: Tailoring Individual Services.

This table is translated from the Norwegian manual (Hatling T, Husum TL, Kjus SHH, Wormdahl I. (2020). [The ReCoN intervention. Strategies to reduce involuntary admissions]. Trondheim: NAPHA - Norwegian Resource Centre for Community Mental Health.)

### Management

Unless there was management ownership it was seen as unlikely that an intervention would succeed. Participants emphasised that management anchoring was crucial for services to be able to prioritise working with the measures as intended. Securing broad commitment within relevant services to prioritise resources, time and service development to support the intervention was seen as crucial. In addition, promoting collaborative agreements with other relevant services like GPs and specialist mental health services could contribute towards this strategy area. Data monitoring was considered an important management tool to detect progress. Since none of the municipalities routinely accessed or reviewed data on involuntary admissions or referrals, this action area included measures to collect and register data. Continuous quality improvement, which is also a management responsibility, was included to promote the structured use of experiences from practice to inform continuous quality improvement. Action areas and measures in this strategy area are listed in Table 2.

### Involving persons with lived experience and family carers

Involving persons with lived experience and family carers in planning and implementing the intervention and individual service provision was seen as central. To achieve this at an organisational level, measures to promote participation in the decision-making, planning and implementation of the intervention were included. At an individual level, post-incident reviews after (referrals to) involuntary admissions and joint crisis plans might facilitate increased participation, in turn strengthening autonomy. Post-incident reviews, following all incidents of (referral to) involuntary admissions can establish individuals’ and family carers’ views and staffs’ experience of the situation and how one can do things differently in the future. Information from such reviews can also be part of the data to inform continuous service improvement under the previous strategy area. Joint crisis plans for those with SMI at risk of involuntary admissions can inform services to intervene early and provide less restrictive alternatives in accordance with individuals’ wishes in situations of mental health deterioration or crisis, and thus prevent involuntary admissions. Stakeholders requested tools/templates for the post-incident reviews and joint crisis plans to help implement these measures in practice. Templates were thus drawn up, taken into the feedback loop, and provided as complementary material to the intervention. The last action area points to the potential of primary mental health services engaging peer workers to take part in the follow-up of persons at risk of involuntary admissions. This can be realised by engaging peer workers already working within the primary mental health services, or the organisation can subsidise new positions. Measures in the strategy area are listed in Table 3.

### Competence development

This strategy area addresses the need for enhanced competency among those working in primary care to better identify and meet the individual needs of those at risk of involuntary admission. Knowledge and increased competence in recovery orientation of services might be particularly important to facilitate the involvement of persons with lived experience and family carers and tailoring individual services as intended in strategy areas 2 and 6. Other areas pointed out as essential training needs included trauma-informed care, the legal framework regulating coercion, assessment of capacity to consent to treatment, knowledge about relevant mental health illnesses (including co-morbidities) and psychiatric medication. Furthermore, open dialogue, motivational interviews, suicide assessment, risk assessment, de-escalation techniques, post-incident reviews, and joint crisis plans also constitute skills and tools that participants perceived might help prevent involuntary admissions if applied by trained staff. Such training might constitute a place where professionals from both primary and specialist care, as well as people with lived experience and family carers, could participate together and facilitate shared understandings. To decide which measures to include in the action area competence-building programme, local needs should be assessed. For their first implementation year, the participants in the current study included measures to enhance competence in assessing capacity to consent to treatment, recovery orientation in mental health services, and trauma-informed care. Measures in the strategy area are listed in Table 4.

### Collaboration across primary and specialist care levels

The strategy area of collaboration across primary and specialist care levels aims to improve collaboration and communication structurally and in individual cases, both prior to, during and following an involuntary admission. Close collaboration between services when assessing the need for involuntary admissions might identify less restrictive alternatives or contribute to finding good solutions if a person is referred but not admitted. Collaboration when assessing an involuntary admission could thus include the assistance of specialist mental health professionals in assessing someone’s capacity to consent to treatment or whether they meet the criteria for admission. Collaboration during and following an involuntary admission includes the joint undertaking in post-incident reviews and the preparation of joint crisis plans described in strategy area 2. In addition, primary mental health services participating in collaboration meetings before an individual is discharged from an involuntary admission can facilitate adequate and individually tailored services in the community at discharge. Joint meeting points, such as evaluation meetings at the management level and responsibility groups at the individual level, might encourage these forms of collaboration. Measures in this strategy area are listed in Table 5.

### Collaboration within the primary care level

Improved collaboration within the primary care level might connect the services better and enhance their collective ability to support those at risk of involuntary admission. Enhanced collaboration between GPs/medical emergency services and the primary mental health services might improve GPs’ or the municipal’s emergency services’ knowledge about which services exist within the local care system. This might help them direct patients towards alternatives to involuntary admissions. Shared meeting points, such as responsibility groups, management meetings, and joint seminars and courses, might facilitate such collaboration, cement relationships and contribute to collective competence development (see strategy area 3). Measures in the strategy area Collaboration within the Primary Care Levels are listed in Table 6.

### Tailoring individual services

In the palm of the hand depicted in Fig. 1, with all the other strategy areas surrounding it, is the Tailoring Individual Services (see Fig. 1). This strategy area aims to promote comprehensive individually tailored recovery-oriented services in close collaboration with the person him or herself by addressing the question “What is important to you?” For most people, a safe home environment is essential and individually tailored accommodation is thus a necessity, but it might also be important to provide a primary care crisis retreat or sheltered housing for those in the early phases of deterioration. Moreover, in order to thrive, support towards a meaningful everyday life might be crucial for many to focus on recovery. This includes finding suitable ways to support individuals to organise their finances, discover meaningful activities, and engage in social networks, as well as getting adequate sleep, a balanced diet, sufficient exercise, and a helpful medication regime. Measures in this strategy area are listed in Table 7.

## Discussion

In this study, researchers and stakeholders developed the ReCoN intervention – a comprehensive intervention for primary mental health care with strategies to reduce involuntary admissions of adults. Dialogue conferences, analysis, and stakeholders’ feedback constituted the co-creation process that directed the ReCoN intervention towards the six strategy areas: 1) Management, 2) Involving Persons with Lived Experience and Family Carers, 3) Competence Development, 4) Collaboration across Primary and Specialist Care Levels, 5) Collaboration within the Primary Care Level, and 6) Tailoring Individual Services. Each strategy area has two to four action areas with measures that constitute the intervention’s practical actions or tasks.

In the discussion of the results, we will, in light of the current literature, address how the ReCoN intervention has the potential to prevent involuntary admissions through its strategies and measures aimed at strengthening individuals’ autonomy and participation, enhancing relevant competence in primary care, and increasing collaboration between services. Furthermore, as we followed a participatory research design with co-creation at its core, we discuss how power imbalances among participants and other aspects of the co-creation process might have influenced the intervention and strengthened or limited its relevance for practice.

### Potential to strengthen individuals’ autonomy and participation

The ReCoN intervention emphasises elements that facilitate the involvement and autonomy of persons with lived experience and their family carers. In the second strategy area, this is emphasised through the *representation at the organisational level*, *joint crisis plan*, and *post-incident review*. To define all these measures as part of one strategy area concerned with involving persons with lived experience and family carers promotes a broad involvement of individuals in service development and individual tailoring of service provision. It emphasises that managers and staff need to involve individuals and let their personal experiences guide service provision. This focus is in line with a recovery-oriented framework, as it supports personally defined recovery, informed choice, and autonomy [18]. Although joint crisis plans have shown effect in reducing involuntary admissions [7, 35–38], they are often not systematically used in current primary mental health services [32]. Increased use of such plans has been shown to have the potential to facilitate shared decision-making processes that balance power inequalities, promote service users’ empowerment, and open up for co-production between individuals and service providers [19]. Studies have found that mental health service users with psychosis who had a plan for early detection and what to do in case of relapse experienced higher support from staff for personal recovery [39]. The inclusion of joint crisis plans in the ReCoN intervention might thus contribute to an intervention effect. In seclusion- and restraint-reduction programs, post-incident reviews have been shown to promote recovery processes, alternatives to seclusion and restraint, improvements in patient care, and organisational development [40]. Whether post-incident reviews developed to use after (referral to) involuntary admissions have the same effect should be explored.

### Potential to enhance relevant competence in primary care

The third strategy area involves competence development in primary health care. Calling the secondary health care level *specialist services* implies that this is where specialised knowledge of SMI has traditionally been present. Primary health care has been more developed towards generalist knowledge. However, as more people with SMI are treated and cared for in the community [15], the need for specialised competence in primary health care increases, as do the public’s expectations of the treatment and care provided at this care level. That many professionals in primary mental health services still believe they need more competence [32] implies specialised knowledge has not been sufficiently provided among staff at this service level. Recovery-oriented and trauma-informed care was among the prioritised competence areas in the ReCoN intervention. These areas are also part of the competence framework in the Six Core Strategies [11], which suggests that these might be essential skills to facilitate across health care levels when aiming to prevent coercive practices.

### Potential to facilitate comprehensive and complementary service provision

The fourth and fifth strategy areas relate to collaboration between services both across care levels (Collaboration Across Primary and Specialist Care Levels) and within the primary care level (Collaboration within the Primary Care Level). Processes leading to involuntary admissions typically unfold in the community and involve multiple services from both primary and specialist care [31]. Poor collaboration and fragmented service provision are factors found both in Norway [41, 42] and other countries [43] that can affect the quality and coherence of service provision to people with SMIs in need of multiple services. Therefore, it was not surprising that the participants in the current study prioritised measures to improve and consolidate collaboration between primary mental health services and other relevant services at both the primary and specialist care levels. The extent of involuntary admissions in the municipality was unknown by professionals working within primary mental health services, suggesting that efforts to prevent such admissions have not been systematically addressed at this care level [32]. By promoting joint efforts and collaborating measures, the ReCoN intervention might facilitate shared focus and effort across services and care levels to prevent involuntary admissions and provide less restrictive alternatives.

### The co-creation process

Relational power imbalances can affect stakeholders’ influence in co-creation processes [21, 44]. For instance, it might be difficult for staff to contradict managers or for people with lived experience to disagree with the psychiatrist from the acute ward at the hospital. The latter might even be difficult for professionals working in primary mental health services who sometimes experience a professional hierarchy where specialist care professionals’ competence is superior to those at primary care [32]. To avoid placing vulnerable individuals in such situations during the co-creation process in the current study, we recruited participants with lived experience and family carers from the local advocacy organisations. Such representation can give an element of empowerment [45]. In addition, in the first group work session, participants were, as far as possible, divided by service, organisation and role (staff/managers) for everybody to feel equal and comfortable to contribute to the dialogue and the brainstorming of measures. Moreover, everybody got the opportunity to individually prioritise the remaining measures with the “star round” at the end of the day.

Different numbers of participants from different stakeholder groups could have given some voices more power than others. For instance, primary mental health services had more participants than specialist mental health services. Similarly, we did not manage to recruit as many participants with lived experience and family carers as hoped. Some groups at the dialogue conferences did not have representatives from all stakeholder groups. Future similar intervention developments should strive to reflect an equal balance of stakeholder groups.

The changes in study design because of the Covid-19 pandemic precluded several stakeholder groups from participating in the feedback loop of the analysis. Instead, the finalisation was made in separate meetings with primary mental health managers, persons with lived experience, and family carers. With this design, the primary mental health managers could influence the last choices about included measures, whereas other professional stakeholder groups, like staff from primary mental health services, staff and managers from secondary mental health services, and primary healthcare medical practitioners, could not. Not gathering all stakeholder groups together might thus have given a result more limited by the primary mental health services’ resources. A sixth dialogue conference with discussions and reflections across stakeholder groups, including people with lived experience and family carers, might have given other perspectives and choices in finalising the intervention. Not including these voices in the finalising stage could have decreased the face validity and acceptability of the intervention result among these stakeholder groups, subsequently affecting implementation.

A participatory research design gives the researchers less control over the research outcome [21, 24]. Still, some choices made by the researchers impact the outcome. In the current study, the researchers defined the research aim and design, planned and facilitated the co-creation process, and gave theoretical input to the participants at the dialogue conferences. All of which affect the co-creation process and might have influenced the participants’ contributions. The researchers’ personal power to influence the results were minimised by having a research team of several researchers with a broad background, including a peer researcher.

### Strengths and limitations

The ReCoN intervention includes measures considered by the participants to be feasible within the resources of current services. Hence, measures like increasing resources, staff, and new municipal housing were excluded during the co-creation process. That the intervention is realistic to carry out within existing resources can strengthen the chance of implementation, but it can also be a potential limitation. McKeown et al. [46] found that insufficient staffing levels hampered efforts to reduce physical restraint. Limited resources, an insufficient staff level, and rigid service allocation in current primary mental health services were factors professionals identified as impeding the prevention of involuntary admissions [32]. Hence, one can assume that the time-consuming nature of the implementation and service development in a comprehensive intervention like the ReCoN intervention might be affected when increased staff level and resources were left out. This can be of particular concern in other contexts where primary mental health care is structured or funded differently than in Norwegian municipalities with publicly funded services with a relatively high staff ratio. The current study’s adaptive approach can, on the other hand, have increased face validity and acceptability, and might have made the intervention more likely to be transferable to practice by strengthening implementation [21] and increasing the facilitation of better service quality [22]. Furthermore, co-creation processes might have established a sense of ownership to the ReCoN intervention in the stakeholders, increasing the chances for implementation and utilisation of the results to practice in the participating municipalities.

The participants were strategically recruited and did not represent a random representative sample. Recruiting through service managers and other key stakeholders in the municipalities could also have given a selection bias as they became gatekeepers for whom they wanted to include in the co-creation process. The high number of participants, and the inclusion of several study sites and multiple stakeholder groups and services, moderated these factors.

The co-creation of the ReCoN intervention was performed in the context of five Norwegian municipalities, and it is not necessarily directly transferable to other settings. However, many of the strategy areas, action areas and measures included in the intervention are related to factors known from the literature to potentially affect involuntary admissions [7, 8, 38]. To increase suitability for an upcoming cluster randomised controlled trial, we developed ReCoN as a consolidated intervention across the participating municipalities. Adapting the measures in the intervention to be eligible to multiple municipalities strengthened its external validity and increased the chances for feasibility elsewhere. However, for some measures, like the competence-building measures, other competency areas might match local needs better. To inform whether competence building measures need to be adapted or can be replicated elsewhere, it would be helpful to explore if workforce development needs are similar or different across different primary mental health care contexts.

The ReCoN intervention includes multiple elements to be implemented in complex contexts at different organisational levels and involving multiple stakeholders. Hence, it is a complex intervention [47, 48] and the assessment of implementation effects and which measures provide which effect is complicated [49]. To compensate for this complexity, we chose a cluster randomised trial design [47], where similar municipalities serve as controls, in an ongoing implementation and feffect study (ClinicalTrials.gov, NCT03989765). This design will enable an outcome for the overall effect but still not assess whether single strategy areas or measures had a larger or smaller effect than others. In addition to testing the effect of the intervention, qualitative implementation monitoring is included. Such qualitative studies can add knowledge on implementation processes, consistency and barriers to change [48]. Developing a fidelity measure could further strengthen future effect assessments and advances.

## Conclusion

There is a lack of comprehensive interventions developed for outpatient contexts aimed to prevent involuntary psychiatric admissions. Thus, the ReCoN intervention developed in the current study has the potential for application to both national and international mental health services. With its full range of stakeholders, the co-creation process strengthens the intervention’s face validity, acceptability, and relevance, making it more likely to be transferable to practice and implemented. Implementation of the ReCoN intervention can increase the focus on and competence in primary mental health care to prevent involuntary admissions and increase the use of less restrictive service alternatives. Furthermore, putting prevention of involuntary admissions on the agenda in primary health care settings has the potential to readdress structurally embedded patterns and promote collaborative efforts to decrease the use of involuntary admissions across health care levels. For persons with SMI, implementing the ReCoN intervention can contribute to fewer experiences of involuntary admissions and that they receive comprehensive services that are recovery-oriented and individually tailored. The effectiveness of the ReCoN intervention is currently being tested in an ongoing cluster randomised controlled trial. Given positive effects, the ReCoN intervention may impact individuals with SMI at risk of involuntary admissions, as more people may experience empowerment and autonomy instead of coercion in their recovery process.

## Data Availability

Data material created in this participatory research study does not have a format suitable for data sharing. Further inquiries can be directed to the corresponding author.

## Abbreviations

SMI: Severe mental illness
ReCoN: Reducing Coercion in Norway
GP: General practitioner
RCT: Randomised controlled trial
